# Assessing the impact of meteorological factors on malaria patients in demilitarized zones in Republic of Korea

**DOI:** 10.1186/s40249-016-0111-3

**Published:** 2016-03-08

**Authors:** Se-Min Hwang, Seok-Joon Yoon, Yoo-Mi Jung, Geun-Yong Kwon, Soo-Nam Jo, Eun-Jeong Jang, Myoung-Ok Kwon

**Affiliations:** Korea Human Resource Development Institute for Health & Welfare, Osong, Republic of Korea; Department of Preventive Medicine, College of Medicine, Korea University, Seoul, Republic of Korea; Department of Preventive Medicine, Armed Forces Medical Command, Seongnam, Republic of Korea; Department of Health Policy and Hospital Management, Graduate School of Public Health, Korea University, 73 Inchon-ro., Seongbuk-Gu, Seoul, 136-705 Republic of Korea; Korea Armed Forces Nursing Academy, 90 Jaun-ro, Yuseong-gu, Daejeon, 305-153 Republic of Korea; Korean Centers for Disease Control and Prevention, Osong, Republic of Korea

**Keywords:** Malaria, *Plasmodium vivax*, Meteorological factors

## Abstract

**Background:**

The trend of military patients becoming infected with *vivax* malaria reemerged in the Republic of Korea (ROK) in 1993. The common explanation has been that infective *Anopheles* mosquitoes from the Democratic People’s Republic of Korea have invaded Republic of Korea’s demilitarized zone (DMZ). The aim of this study was to verify the relationship between meteorological factors and the number of malaria patients in the military in this region.

**Methods:**

The authors estimated the effects of meteorological factors on *vivax* malaria patients from the military based on the monthly number of malaria cases between 2006 and 2011. Temperature, precipitation, snow depth, wind velocity, relative humidity, duration of sunshine, and cloud cover were selected as the meteorological factors to be studied. A systematic pattern in the spatial distribution of malaria cases was assessed using the Moran’s Index. Granger causality tests and cross-correlation coefficients were used to evaluate the relationship between meteorological factors and malaria patients in the military.

**Results:**

Spatial analysis revealed significant clusters of malaria patients in the military in Republic of Korea in 2011 (Moran’s I = 0.136, *p*-value = 0.026). In the six years investigated, the number of malaria patients in the military in Paju decreased, but the number of malaria patients in the military in Hwacheon and Chuncheon increased. Monthly average, maximum and minimum temperatures; wind velocity; and relative humidity were found to be predicting factors of malaria in patients in the military in Paju. In contrast, wind velocity alone was not able to predict malaria in Hwacheon and Chuncheon, however, precipitation and cloud cover were able to predict malaria in Hwacheon and Chuncheon.

**Conclusions:**

This study demonstrated that the number of malaria patients in the military is correlated with meteorological factors. The variation in occurrence of malaria cases was principally attributed to differences in meteorological factors by regions of Republic of Korea.

**Electronic supplementary material:**

The online version of this article (doi:10.1186/s40249-016-0111-3) contains supplementary material, which is available to authorized users.

## Multilingual abstracts

Please see additional file 1 for translations of the abstract into the six official working languages of the United Nations.

## Background

Malaria is a major cause of illness and death in children and adults worldwide. An estimated 300–500 million clinical cases of malaria are reported each year [1–3]. Changing weather patterns are risk factors for the disease [4–6].

*Plasmodium vivax*, the causative agent of malaria in Korea, was endemic to the Korean Peninsula until the late 1970s, when the country was declared malaria free [7]. However, malaria was detected again in a soldier who was serving in Paju, in the western part of the demilitarized zone (DMZ), in 1993 [8]. Since then, the annual incidence rate of *vivax* malaria has increased, with more than 2000 cases reported by the end of 1997 [9]. During this period, malaria had largely been confined to the northern part of Gyeonggi Province and the northwestern part of Gangwon Province near the DMZ areas, where there is a high concentration of soldiers [10]. The number of annual *vivax* malaria cases, which had increased rapidly during 2000, marked also by a geographic expansion, started to decrease in 2001, reducing to 864 cases in 2004. The trend, however, turned around again in 2005, when 1304 cases were reported [11]. The number of cases rose by 54.0% in 2006, but the rate of increase slowed down in 2007 [12].

The most popular opinion is that malaria reemerged in Republic of Korea because of a continuous influx of infective mosquitoes from the Democratic People’s Republic of Korea, where malaria has been persistent [13, 14]. Another argument is that an outbreak of *vivax* malaria in Korea in the 1990s is a relapse from activated hypnozoites among Korean soldiers [15]. The purpose of this study was to examine the origin of reemerging malaria in patients in the military, as well as the relationship between meteorological factors and malaria patients in the military in certain areas surrounding the DMZ.

## Methods

### Subjects and study sites

**Fig. 1:**
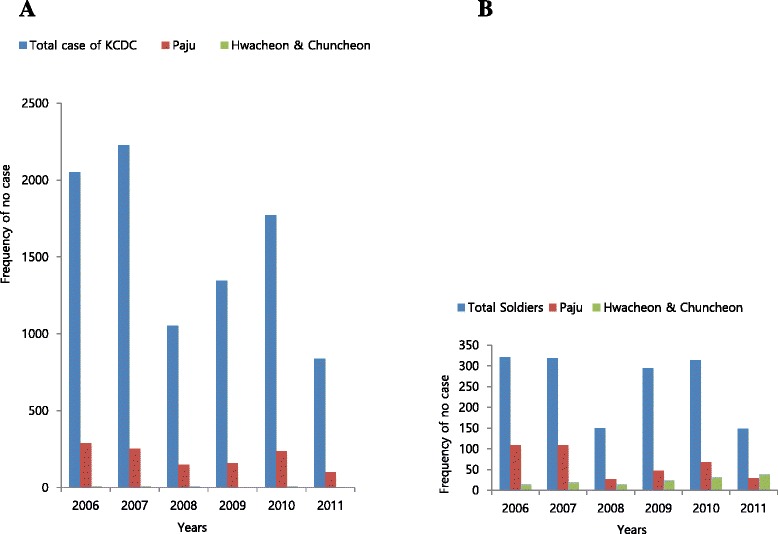
Geographical distribution of malaria patients in South Korea from 2006 to 2011 ((**a**): total cases, data from the KCDCs; (**b**): malaria patients in the military, data from the AFMC). B*: Total military malaria cases were 321 in 2006, and the distribution of military malaria cases (N ≧ 10) in 2006 is: 108 (33.6%) in Paju, Gyeonggi-do; 82 (25.6%) in Dongducheon, Gyeonggi-do; 56 (17.5%) in Cheorwon, Gangwon-do; 15 (4.7%) in Hwacheon and Chuncheon, Gangwon-do; 13 (4.1%) in Incheon; and 11 (3.4%) in Inje, Gangwon-do. The case number reduced to 148 cases in 2011 and the distribution of military malaria cases (N ≧ 10) in 2011 is: 39 (26.4%) in Hwacheon and Chuncheon, Gangwon-do; 39 (26.4%) in Cheorwon, Gangwon-do; 35 (23.7%) in Dongducheon, Gyeonggi-do; 29 (19.6%) in Paju, Gyeonggi –do; 4 (2.7%) in Inje, Gangwon-do; and 4 (2.7%) in Incheon

The authors obtained surveillance data of civilian malaria cases, including dates of onset and places of residence, from the Korea Centers for Disease Control and Prevention (KCDCs; Osong, ROK), and from the Armed Forces Medical Command (AFMC; Seongnam, ROK) for malaria patients in the military. Doctors passively surveying malaria patients in the military gave us our data. According to identified dynamics and diversity of topographical features, two out of 228 weather station areas across the country, Paju (Munsan) and Chuncheon (Hwacheon and Chuncheon), were selected for the study because these two areas showed the most significant changes in the numbers of malaria patients in the military between 2006 and 2011. The largest decrease in the number of malaria patients in the military was observed in Paju, Gyeonggi Province, at 37°54’ north latitude and 127°44’ east longitude; while the biggest increase in the number of malaria patients in the military was observed in Chuncheon, Gangwon Province, at 37°53’ north latitude and 126°45’ east longitude (see B* in Fig. 1).

### Meteorological data

Monthly meteorological records from 2006 to 2011 were acquired from the Korea Meteorological Administration (Seoul, ROK). The meteorological data included average, maximum, and minimum temperatures; precipitation; snow depth; wind velocity; relative humidity; duration of sunshine; and cloud cover. The data were collected and recorded by the local weather station in each area (Munsan and Chuncheon). The Munsan weather station is located in Paju (37°53’ north latitude, 126°45’ east longitude, and 30 meters in altitude). The Chuncheon weather station monitors both Chuncheon and Hwacheon (37°53’ north latitude, 126°44’ east longitude, and 76.82 meters in altitude). These meteorological variables and the number of malaria cases were used to estimate the effect of meteorological factors on patients with *P. vivax* malaria [16]*.*

### Statistical analysis

We used Spearman’s rank correlation coefficients to examine the relationship between the annual number of malaria patients and meteorological variables using SPSS software (version 12.0; SPSS Inc., Chicago, IL, USA), as the sample size was small and the data were not normally distributed. The longitudes and latitudes of the studied areas were prepared using GeoMedia Professional version 6.1 (Intergraph, USA). Data was migrated to R software (version 3.0.3; R Project for Statistical Computing, Seoul, Korea) for further analysis. Spatial analysis was performed using the software R version 3.0.3 to calculate the Moran’s Index for clusters of malaria patients in the military [17, 18]. A Granger causality test was carried out in order to assess whether there was any potential predicting power of meteorological factors on malaria patients in the military, using SAS 9.3 for Windows (SAS Institute, Inc., Cary, NC, USA) [19–21]. The Granger causality test requires that the time series are covariance stationary, so an Augmented Dickey-Fuller test was also performed. For all if the series the null hypothesis *H*_0_ of non-stationarity can be rejected at a 5% confidence level; In other words, if the Dickey-Fuller Unit Root test with a *p*-value < 0.05, then the meteorological time series also have standard distributions. Therefore, we can use the Granger causality test [22].

### Ethics statement

Data on malaria patients were provided by the KCDCs and AFMC, and extracted from annual reports, which provided summarized count data of patients classified by county and month. All data were anonymized. The local institutional review board at the AFMC approved the study protocol. The Institutional Review Board number was AFMC-11-IRB-020.

## Results

### Frequency of malaria and distribution of malaria patients in the military in Republic of Korea from 2006 to 2011

In 2006, a total of 2051 malaria cases were reported by the KCDCs and 321 malaria cases in the military were reported by the AFMC. The Munsan weather station reported the highest number of patients in Paju (KCDC: 288 patients, AFMC: 108 patients). In stark contrast, only 20 civilian and 15 military malaria cases were reported by the Chuncheon weather station in 2006. Although there was a mixed trend in the number of malaria patients reported in Paju, there has been a constant decline in the area since 2006, with as few as 29 malaria patients in the military being reported in 2011, the lowest number during the given period. However, the number of malaria patients in the military stationed in the Chuncheon weather station area steadily increased from two in 2006 to 39 in 2011, thus becoming the most vulnerable area for malaria (see Fig. 1).

Malaria patients in the military were all males aged 18 to 53 (22.33 ± 2.99). Out of 531 military patients infected from 2006 to 2011, the incidence rates by ranks were as follows: 238 (44.8%) corporals, 145 (27.3%) sergeants, 39 (7.3%) staff sergeants, 38 (7.2%) privates first class, 29 (5.5%) sergeants first class, 20 (3.8%) first lieutenants, 12 (2.3%) privates, and 10 (1.9%) of other ranks. Relapse/reinfection in malaria patients in the military was reported up to 12 months (3.15 ± 3.98) after the first treatment. The patients took a total of 2000 mg of hydroxychloroquine (HCQ, Korean United Pharmaceutical Inc. Seoul, Republic of Korea): 800 mg at the point of diagnosis, and 400 mg doses at six, 24, and 48 hours after diagnosis. Then, they took 15 mg of primaquine (Myung In Pharmaceutical, Seoul, ROK) daily for the following 14 days. There were only 26 (4.9%) relapsed/reinfected cases out of 531 patients from 2006 to 2011. In addition, of those 26 patients, 19 (73.08%) were from army divisions adjacent to the DMZ.

### Spearman’s rank correlation between the number of malaria patients and meteorological factors

A strong correlation was observed between the number of civilian and military malaria patients and minimum temperature (*r* = 0.771, *p* < 0.1) (Table 1). As well as that, positive or negative relationships were observed between the number of civilian and military malaria patients (*r* = 0.943, *p* < 0.01), and the average and maximum temperatures (*r* = 0.771, *p* < 0.1), the average and minimum temperatures (*r* = 0.771, *p* < 0.1), the maximum temperature and precipitation (*r* = -0.829, *p* < 0.01), and precipitation and duration of sunshine (*r* = -0.771, *p* < 0.1).

**Table 1:** Spearman rank correlation between malaria patients and annually average meteorological factor of South Korea (2006–2011)

	Civilians	Soldiers	Average temperature	Maximum temperature	Minimum temperature	Precipitation	Snow depth	Wind velocity	Relative humidity	Duration of sunshine	Cloud amount
Civilians	1.00	0.943***	0.543	0.086	0.771*	0.086	0.086	0.257	0.486	−0.429	0.429
Soldiers	0.943***	1.00	0.371	0.029	0.714	−0.029	0.143	0.429	0.543	−0.200	0.371
Average Temperature	0.543	0.371	1.00	0.771*	0.771*	−0.371	−0.143	−0.429	0.143	−0.086	0.314
Maximum temperature	0.086	0.029	0.771*	1.00	0.543	−0.829**	0.200	−0.543	0.029	0.486	−0.029
Minimum temperature	0.771*	0.714	0.771*	0.543	1.00	−0.257	0.200	−0.314	0.714	0.029	−0.029
Precipitation	0.086	−0.029	−0.371	−0.829**	−0.257	1.00	−0.371	0.143	0.086	−0.771*	0.029
Snow depth	0.086	0.143	−0.143	0.200	0.200	−0.371	1.00	−0.086	0.371	0.314	−0.486
Wind velocity	0.257	0.429	−0.429	−0.543	−0.314	0.143	−0.086	1.00	−0.257	−0.200	0.600
Relative humidity	0.486	0.543	0.143	0.029	0.714	0.086	0.371	−0.257	1.00	0.143	−0.543
Duration of sunshine	−0.429	−0.200	−0.086	0.486	0.029	−0.771*	0.314	−0.200	0.143	1.00	−0.486
Cloud amount	0.486	0.543	0.143	0.029	−0.029	0.029	−0.486	0.600	−0.543	−0.486	1.00

* *P*-value < 0.1, ** *P*-value < 0.05, *** *P*-value < 0.01

### Spatial distribution of malaria patients in Republic of Korea

**Fig. 2:**
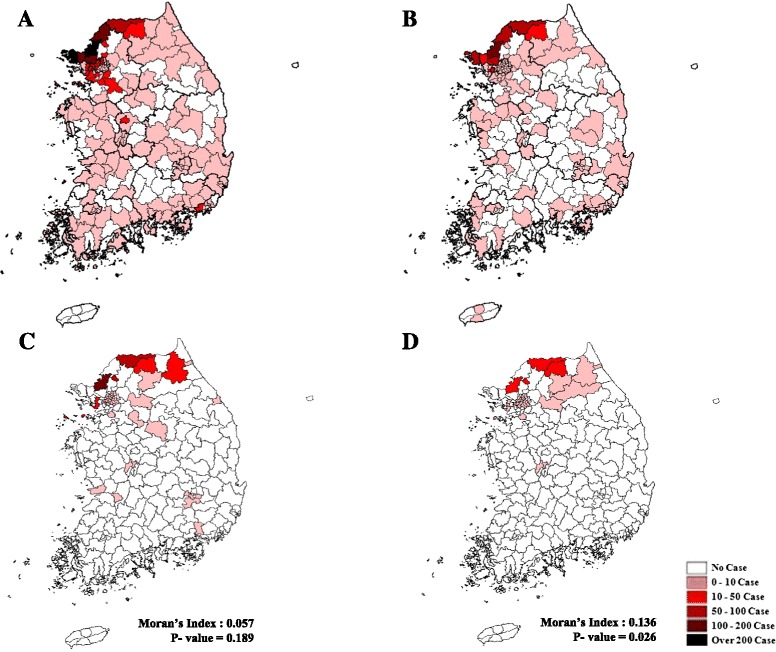
Spatial distribution of malaria patients in South Korea. **a**: civilian malaria patients, 2006; (**b**): civilian malaria patients, 2011; (**c**): malaria patients in the military, 2006; (**d**): malaria patients in the military, 2011. * Cluster pattern of malaria patients in the military was estimated using the Moran’s Index (**c**, **d**)

As seen in Fig. 2, in 2006, malaria seemingly broke out across the nation even though the cases were concentrated in the northwestern part of the 38^th^ parallel, which separates the Democratic People’s Republic of Korea and Republic of Korea, with the DMZ as a buffering zone. On the other hand, the epidemic in 2011 did not affect many areas of the country, but a high occurrence rate was still seen in locations around the parallel. In terms of a malaria outbreak in the military, cases were mainly reported from areas near the 38^th^ parallel, and some from other parts of the country. However, no significant clusters were identified (Moran’s I = 0.057, *p*-value = 0.189). In 2011, there were hardly any malaria patients in the military in areas other than those surrounding the DMZ. Additionally, the number of malaria cases drastically decreased in Paju, where the highest number of cases was reported in 2011; while the highest numbers of cases with a significant cluster (Moran’s I = 0.136, *p*-value = 0.026) were observed in Hwacheon, Chuncheon, and Cheorwon, in the eastern part of the country and relatively close to the DMZ.

### Times-series plots for monthly malaria patients in the military

**Fig. 3:**
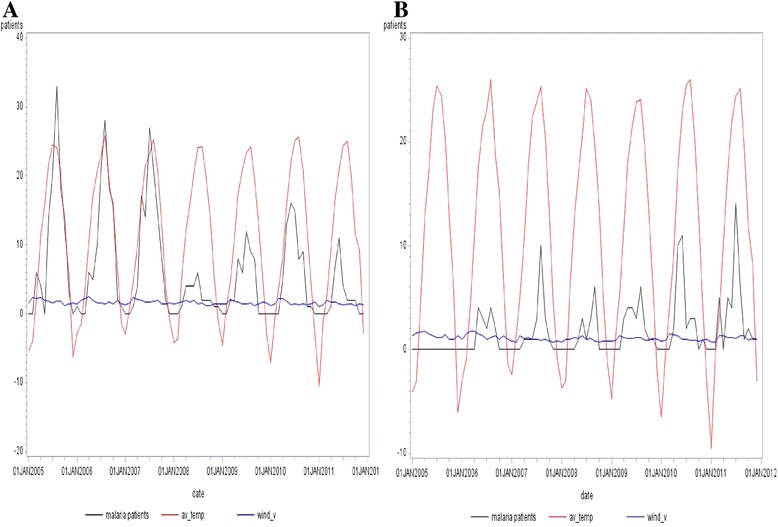
Times-series plots for monthly malaria patients in the military, using wind velocity as a variable (Jan 2006 – Dec 2011). *(**a**): Paju, (**b**): Hwacheon and Chuncheon. av_temp: average temperature, wind_v: wind velocity

Over the entire period of the study (Jan 2006 – Dec 2011), a strong seasonal trend was observed in outbreaks of malaria in patients in the military: the largest number of patients was reported in Paju, and in Hwacheon and Chuncheon in summer, when the average temperatures were the highest. The two areas showed similar monthly maximum temperatures on average. The only difference was that the size of waves of malaria patients in the time-series plots drastically decreased from 2008 in Paju, while they gradually increased in the Chuncheon weather station area (see Fig. 3).

### Stationarity around a linear time trends by unit root test

According to the Augmented Dickey-Fuller Test, both for Munsan and Chuncheon, no unit root was identified between the number of malaria patients in the military and the variables of average, maximum, and minimum temperatures; precipitation; snow depth; wind velocity; relative humidity; and duration of sunshine (*p*-value < 0.01). The only unit root identified was cloud cover in Munsan (*p*-value = 0.7687) (Table 2). Based on these findings, the authors carried out a Granger causality test to verify the impact of meteorological factors on the number of malaria patients in the military in the two areas. The results are described in the following section.

**Table 2:** Unit root tests of military malaria patients and meteorological factors of South Korea (2006–2011)

	Munsan weather station		Chuncheon weather station	
	DF*	*p*-value	DF*	*p*-value
Military malaria patients	−4.48	0.0029**	−5.26	0.0002**
Average temperature	−7.43	<0.0001**	−8.04	<0.0001**
Maximum temperature	−7.54	<0.0001**	−7.58	<0.0001**
Minimum temperature	−6.83	<0.0001**	−7.28	<0.0001**
Precipitation	−5.72	<0.0001**	−5.99	<0.0001**
Snow depth	−4.88	0.0008**	−6.24	<0.0001**
Wind velocity	−5.90	<0.0001**	−4.26	0.0058**
Relative humidity	−4. 94	0.0006**	−4.57	0.0022**
Duration of sunshine	−6.30	<0.0001**	−5.80	<0.0001**
Cloud amount	−1.64	0.7687	−4.69	0.0015**

* *P*-value of Trend is calculated by the Dickey-Fuller Unit Root Tests of 1^st^ difference

** *P*-value < 0.01

### Results of the Granger causality test

As shown in Table 3, average, maximum, and minimum temperature; wind velocity; and relative humidity affected the monthly number of malaria patients in the military in Paju by time lag. The average and maximum temperatures had significant causal effects on the number of patients in nine time lags out of 12, and wind velocity had significant causal effects on the number of patients in nine consecutive time lags from Lag 1 to 9. There were just two significant Granger causalities, Lag 2 and 5, for relative humidity.

**Table 3:** Results of Granger causality tests of military malaria patients and meteorological factors of South Korea (2006–2011)

Weather station	meterological factor	*P*-value of Granger causality tests for military malaria patients and meteorological factors by time lag*											
		Lag1	Lag2	Lag3	Lag4	Lag5	Lag6	Lag7	Lag8	Lag9	Lag10	Lag11	Lag12
Munsan (Paju)	Average temperature	0.1552	**0.0020**	0.0689	**0.0018**	**0.0004**	**0.0194**	0.0526	**0.0271**	**0.0105**	**0.0409**	**0.0281**	**0.1051**
	Maximum temperature	0.1836	**0.0075**	0.0804	**0.0002**	**0.0001**	**0.0062**	**0.0212**	**0.0111**	**0.0109**	**0.0499**	**0.0225**	0.0842
	Minimum temperature	0.1971	**0.0021**	0.0675	**0.0101**	**0.0021**	0.0670	0.1252	**0.0479**	**0.0165**	**0.0570**	**0.0412**	0.2024
	Precipitation	0.7640	0.4208	0.9007	0.5159	0.4214	0.3370	0.2346	0.2490	0.7613	0.8176	0.6669	0.3958
	Snow depth	0.5503	0.6724	0.5912	0.1347	0.1493	0.4483	0.4807	0.5974	0.6531	0.8632	0.8522	0.8981
	Wind velocity	**0.0027**	**0.0004**	**0.0021**	**<0.0001**	**<0.0001**	**<0.0001**	**<0.0001**	**<0.0001**	**0.0212**	0.0823	0.1809	0.3732
	Relative humidity	0.1999	**0.0204**	0.1022	0.0876	**0.0056**	0.0563	0.2070	0.1778	0.7396	0.7500	0.5309	0.6492
	Duration of sunshine	0.8989	0.3776	0.4425	0.6541	0.6931	0.4450	0.5569	0.3409	0.6096	0.5021	0.1100	0.0784
	Cloud amount	0.7208	0.9018	0.7702	0.8680	0.8493	0.8807	0.9605	0.9686	0.9888	0.9932	0.9678	0.8256
Chuncheon (Hwacheon & Chuncheon)	Average temperature	0.0550	**0.0010**	**0.0041**	**0.0003**	**0.0031**	**0.0025**	**0.0008**	**0.0010**	**0.0027**	**0.0077**	**0.0401**	0.1715
	Maximum temperature	0.0583	**0.0025**	0.0086	**0.0010**	**0.0042**	**0.0046**	**0.0020**	**0.0021**	**0.0058**	**0.0170**	0.0732	0.2441
	Minimum temperature	0.0843	**0.0008**	**0.0033**	**0.0002**	**0.0026**	**0.0016**	**0.0005**	**0.0005**	**0.0015**	**0.0048**	**0.0285**	0.1447
	Precipitation	**0.0150**	**0.0073**	**0.0186**	**0.0264**	**0.0496**	0.1082	**0.0391**	**0.0194**	**0.0273**	**0.0100**	0.0511	0.1903
	Snow depth	0.1237	0.2803	0.1989	0.1360	0.1450	0.1084	**0.0156**	**0.0362**	**0.0116**	**0.0101**	**0.0272**	**0.0468**
	Wind velocity	0.1681	0.2643	0.4348	0.1426	0.1650	**0.0121**	**0.0078**	**0.0131**	0.0605	0.1219	0.3410	0.6275
	Relative humidity	0.3833	**0.0109**	**0.0255**	**0.0193**	**0.0343**	**0.0470**	0.0595	**0.0318**	0.0536	0.0554	0.1133	0.3923
	Duration of sunshine	0.6006	0.2905	0.4142	0.3966	0.5688	0.7007	0.7048	0.8026	0.8879	0.9511	0.9903	0.9995
	Cloud amount	**0.0269**	**0.0418**	**0.0144**	**0.0220**	0.0704	0.0630	0.2035	0.2135	0.1789	0.1825	0.2884	0.6158

* *P*-value < 0.05

For Chuncheon weather station area, the average, maximum, and minimum temperature; precipitation; relative humidity; and cloud cover all had an influence on the number of malaria patients in the military. Average, maximum, and minimum temperatures, and precipitation demonstrated significant causal relationships with the monthly census of malaria cases in nine to ten time lags each. Relative humidity and cloud cover had statistically significant impacts on the number of patients in six and four time lags, respectively.

### Results of cross-correlation

Cross-correlation coefficients were analyzed to observe the relationship between the monthly number of malaria patients in the military and meteorological factors (Table 4). In Paju, average temperature (Lag 2, cross-correlation coefficients: 0.35155), maximum temperature (Lag 2, cross-correlation coefficients: 0.34596), minimum temperature (Lag 2, cross-correlation coefficients: 0.34165), and wind velocity (Lag 1, cross-correlation coefficients: 0.2284; Lag 2, cross-correlation coefficients: 0.44321; Lag 3, cross-correlation coefficients: 0.52579; Lag 4, cross-correlation coefficients: 0.61399; Lag 5, cross-correlation coefficients: 0.44778; Lag 6, cross-correlation coefficients: 0.25446) were found to be significantly correlated with the number of malaria patients.

**Table 4:** Results of Cross-correlation of military malaria patients and meteorological factors of South Korea (2006–2011)

Weather station	meterological factor	Cross-correlation coefficients of military malaria patients and meteorological factors by time lag											
		Lag1	Lag2	Lag3	Lag4	Lag5	Lag6	Lag7	Lag8	Lag9	Lag10	Lag11	Lag12
Munsan (Paju)	Average temperature	0.57974	**0.35155**	0.04358	−0.29142	−0.5619	−0.64975	−0.55559	−0.29692	0.03139	0.30964	0.49777	0.52463
	Maximum temperature	0.56266	**0.34596**	0.05285	−0.28175	−0.55878	−0.65644	−0.55664	−0.29270	0.03924	0.30936	0.49574	0.51516
	Minimum temperature	0.58073	**0.34165**	0.02129	−0.30964	−0.56946	−0.64196	−0.54372	−0.29042	0.03367	0.31436	0.50068	0.52804
	Precipitation	0.30709	0.12049	−0.05356	−0.26302	−0.36700	−0.35330	−0.31283	−0.19622	−0.02474	0.18855	0.24974	0.33081
	Snow depth	−0.29098	−0.21836	−0.06296	0.1905	0.3665	0.40857	0.25944	0.12656	−0.11301	−0.20772	−0.29325	−0.31238
	Wind velocity	**0.22814**	**0.44321**	**0.52579**	**0.61399**	**0.44778**	**0.25446**	−0.07253	−0.2131	−0.31132	−0.26316	−0.17564	−0.06557
	Relative humidity	0.36669	0.05854	−0.26161	−0.4935	−0.61343	−0.54843	−0.33246	−0.07683	0.20975	0.41969	0.48565	0.44976
	Duration of sunshine	−0.25824	−0.07013	0.10738	0.19772	0.19205	0.09003	0.09689	0.08826	0.02436	−0.09703	−0.0727	−0.14867
	Cloud amount	−0.09007	−0.09011	−0.10937	−0.10022	−0.12033	−0.0779	−0.05642	−0.01894	−0.00161	0.00932	0.02754	0.03294
Chuncheon (Hwacheon & Chuncheon)	Average temperature	0.40453	**0.17028**	−0.08712	−0.36101	−0.52516	−0.55360	−0.43391	−0.18914	0.11511	0.37533	0.52307	0.54965
	Maximum temperature	0.39775	**0.17289**	−0.07968	−0.35042	−0.52654	−0.55914	−0.43723	−0.19351	0.11127	0.36997	0.51275	0.54316
	Minimum temperature	0.38967	**0.14221**	−0.11379	−0.38639	−0.52500	−0.53871	−0.41130	−0.16382	0.13709	0.39590	0.53562	0.54655
	Precipitation	**0.42634**	0.07917	**−0.10708**	**−0.22790**	**−0.28368**	−0.25436	−0.25591	−0.18232	0.04048	0.34549	0.35692	0.26451
	Snow depth	−0.27816	−0.15307	0.06974	0.26459	0.33595	0.16506	0.22384	0.10788	−0.17168	−0.21546	−0.30791	−0.29028
	Wind velocity	0.14029	0.21942	0.17884	−0.01403	−0.14410	−0.34594	−0.41934	−0.38530	−0.29720	−0.20910	−0.09502	0.02567
	Relative humidity	0.07506	**−0.20981**	**−0.32562**	**−0.40541**	**−0.25653**	**−0.11512**	0.04494	0.23698	0.38670	0.49291	0.43361	0.27622
	Duration of sunshine	0.04313	0.17192	0.18042	0.21115	0.01058	−0.09447	−0.09895	−0.11490	−0.13595	−0.15572	−0.08456	0.03621
	Cloud amount	**0.38957**	**0.19240**	−0.00265	−0.25102	−0.27639	−0.34421	−0.33002	−0.16087	0.04080	0.27608	0.41410	0.39907

In the Chuncheon weather station area, the significant variables were average temperature (Lag 2, cross-correlation coefficients: 0.17028), maximum temperature (Lag 2, cross-correlation coefficients: 0.17289), minimum temperature (Lag 2, cross-correlation coefficients: 0.14221), precipitation (Lag 1, cross-correlation coefficients: 0.42634), relative humidity (Lag 2, cross-correlation coefficients: -0.20981; Lag 3, cross-correlation coefficients: -0.32562; Lag 4, cross-correlation coefficients: -0.40541; Lag 5, cross-correlation coefficients: -0.25653; Lag 6, cross-correlation coefficients: -0.11512), and cloud cover (Lag 1, cross-correlation coefficients: 0.38957; Lag 2, cross-correlation coefficients: 0.19240).

### Dynamics of malaria patients in military bases

**Fig. 4:**
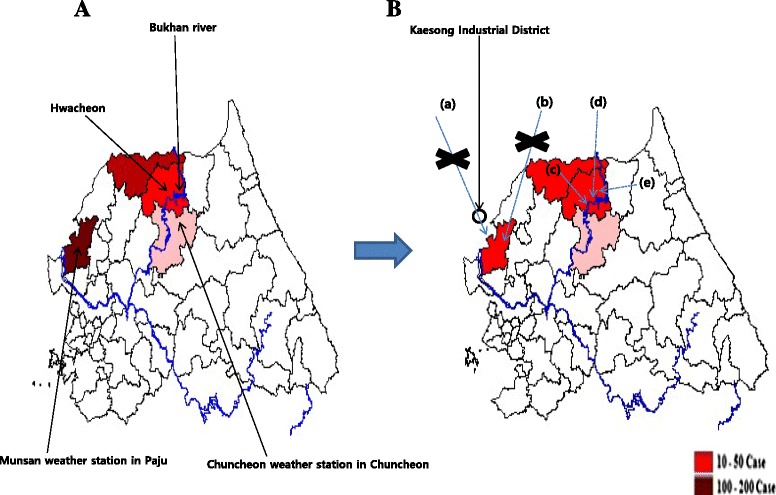
Dynamics of malaria outbreaks in the military: comparison of Paju and the Chuncheon weather station area. **a**: malaria patients in the military, 2006. **b**: malaria patients in the military, 2011. (a) Kaesong Industrial Complex began operating at the beginning of 2007. As a result, there might have been an influx of mosquitos carrying *P. vivax* malaria from north DMZ to Paju. (b), (d) Paju had been heavily influenced by northeasterly wind from 2006 to 2007, but its effects weakened between 2008 and 2011. As a result, transmission of *P. vivax* malaria by mosquitos from north DMZ to Paju might be contained. (c), (e) As Bukhan River might be a mosquito larvae reservoir, this might contribute to an increase in the number of malaria patients in the military around this area: malaria patients decreased in Cheorwon (c), and Inje (e), but otherwise malaria patients in the military increased in the Chuncheon weather station area (Hwacheon and Chuncheon)

The geographical characteristics, and geopolitical and meteorological changes both in Paju and the Chuncheon weather station area are presented in Fig. 4. There are a few prominent features in both areas. The Chuncheon weather station area is near the Bukhan River, which crosses the area and serves as a mosquito larvae reservoir. Paju is close to the Kaesong Industrial Districts that were built in 2007 in the northwestern part of the DMZ. The industrial complex acts as a windshield that hinders mosquitos from fleeing into south of DMZ.

## Discussion

The findings of this study revealed that ROK military personnel serving in the region near the DMZ were susceptible to malaria infection between 2006 and 2011, with meteorological factors playing an important role in the epidemic. Ups and downs were observed in the trend of malaria outbreaks in the civilian population, as well as in the military, as reported by the KCDCs and AFMC, respectively, with the highest numbers of malaria patients reported in 2006 and 2007, and the lowest in 2011, by both institutions. This result implies that incidence of malaria in the civilian and military populations is affected by the same factors.

Of all the meteorological variables, the overall number of malaria patients was significantly correlated only with minimum temperature. This result is inconsistent with a study by Kim and Jang, who also studied malaria in Republic of Korea from 2001 to 2008 [23]. They reported that the number of malaria patients in the country was significantly correlated with average, maximum, and minimum temperatures; relative humidity; and precipitation (*r* > 0.8, *p* < 0.05). Unlike their study, this study found that minimum temperature has a significant correlation with the number of malaria cases (*r* = 0.77, *p* < 0.1). This difference could be explained by the fact that Kim and Jang studied the relationship between monthly meteorological data and the number of patients, while this study analyzed the relationship between the averages of each meteorological factor over six years and the number of patients, which might have caused statistical significance to disappear. In other words, ecological fallacy, often seen in ecological studies, also happened in this study [24]. To avoid this, the authors should have divided the area into smaller parts and chosen monthly meteorological factors for analysis. Lena Hulden and colleagues reported that, despite the error mentioned above, there was a low chance of *Anopheles sinensis* mosquitoes, vectors of malaria, flying into Republic of Korea as the wind mainly blows from south to north in the summer [15]. However, the directions and velocity of wind vary upon seasons and regions. Therefore, it is necessary to take geographical characteristics into consideration when studying the relationship between meteorological factors and malaria in order to arrive at more conclusive findings.

To offset the fallacy in this study, patient clusters were verified with data from 2006, when the number of malaria patients was the highest, and from 2011, when it was the lowest, to observe the regional distribution of malaria patients. As well as that, the Granger causality and cross-correlation tests were performed to verify the relationship between the number of malaria patients in the military and meteorological factors in Paju, where the first malaria patient in the military was recorded in 1993 [8], and in the Chuncheon weather station area, the only place where the number of malaria patients in the military continuously increased from 2006 to 2011. The authors of this study found that the clustering tendency of malaria cases in the military was significantly stronger in the DMZ area in 2011 than it was in 2006. The pattern of malaria outbreaks between 1993 and 2000 was that the distribution of patients increasingly expanded to the eastern part of the 38^th^ parallel from Paju, on the western part of the line [10, 25]. Additionally, domestic studies have reported that the number of malaria patients in the military rapidly increased from 159 in 2004 to 445 in 2007 [11, 25]. In this study, the Moran’s Index, representing clustering tendency, significantly increased in neighboring areas of the DMZ as the number of malaria patients in the military decreased. By contrast, the Index decreased with no statistical significance when the range of distribution was expanded beyond the DMZ and the number of malaria cases increased.

Regarding the relationship between meteorological factors and the number of malaria patients in the military, average humidity was the highest in 2006 when the number of malaria patients in the military reached a record high during the study period. On the other hand, in 2011 (during which had the lowest number of malaria patients was recorded), the lowest average, minimum, and maximum temperatures and the highest precipitation were recorded. These findings lead to the conclusion that the lowest annual average, minimum, and maximum temperatures correlate with the lowest number of malaria patients, and that high humidity correlates with the highest number of malaria patients. This is because higher temperatures promote breeding and the proliferation of *A. sinensis* mosquitoes, thus extending the length of the malaria transmission season [26, 27]. In addition, high humidity increases the vector’s survival rate [28]. A higher annual precipitation generally contributes to a larger number of larva habitats, meaning that the number of malaria patients also grows. However, the lowest number of malaria patients was recorded in 2011, when the precipitation was also the highest, but this was due to a typhoon and flooding, which consequently swept away the habitats and vectors themselves.

According to the results of the time-series analysis, the average, maximum, and minimum temperatures with a two-month time lag, and wind velocity with one- to six-month time lags were significantly and positively correlated with the number of malaria cases in Paju, when the results of the Granger causality and cross-correlation coefficient tests were combined. Based on these findings, it can be suggested that *A. sinensis* mosquitoes flew from the north or the northern part of DMZ into Paju along with the wind, or following the onset of the appropriate temperature to breed, resulting in a resurgence of malaria, which was thought to have disappeared. In the Chuncheon weather station area, temperature, precipitation, relative humidity, and cloud cover affected the outbreak of malaria with one- to six-month time lags. More specifically, the average, maximum, and minimum temperatures with a two-month time lag, and precipitation with a one-month time lag were positively correlated with an increase in the number of malaria patients. On the other hand, precipitation and relative humidity with two- to six-month time lags were negatively correlated with the number of malaria patients. Because Bukhan River, which is part of the Chuncheon weather station area, serves as an *A. sinensis* mosquito larvae reservoir, increase in precipitation destroys their habitats, which thus leads to a decrease in the number of malaria patients. Other studies reported similar results. For example, as the distance from the Karanga River in northern Tanzania increases by 50 meters, the number of malaria patients decreases (OR = 0.96, 95% CI: 0.92–0.998) [29]. Within a distance of 60 meters from the Huang-Huai River in central China, the risk of developing malaria is 1.6 times (95% CI: 1.042–2.463) higher [30]. These results imply that if larva habitats were washed away by rising precipitation in a river, the probability of a malaria outbreak would decrease. Cloud cover had a positive correlation with the number of malaria patients in the military, with about one- to two-month time lag. This finding is consistent with the findings of a study in Mengla County of southwest China, which reported that the frequency of foggy days positively affected the number of malaria patients. An increase in cloud cover and foggy day frequency is considered to improve conditions for aquatic breeding [31].

Other factors besides meteorological ones influenced the number of malaria patients. For instance, as an industrial complex was built in 2007 in Kaesong, The Democratic People’s Republic of Korea, in the north of the DMZ, near Paju, preventive actions against vector-borne diseases including malaria were strengthened, and part of the forest around the industrial district was cleared. Subsequently, the rate of influx of *A. sinensis* mosquitoes into Paju and the probability of contact with them reduced. Furthermore, *A. sinensis* mosquitoes living around Paju and the DMZ area were likely to move to Bukhan River in the Chuncheon weather station area because of the temperature, direction of the wind, and changes in the environment around the Kaesong Industrial Complex, which led to an increased number of malaria patients around Chuncheon. On the other hand, Lena Hulden argued that the actual cause of malaria prevalence in Korean soldiers stationed in areas close to the 38^th^ parallel was relapses from activated hypnozoites due to the malaria incidence rate in the soldiers being six to seven times higher than those of US soldiers deployed in the same area between 1993 and 2005 [15]. Noteworthy and relevant to the discussion of the high infection rate in Korean soldiers is that they were enrolled by the conscription system and worked 24 hours a day in shifts, living in barracks. In contrast, the US soldiers, who were volunteers, usually stayed in their official residencies outside bases, except for some who worked in Panmunjom, a village built on the de facto border between the Democratic People’s Republic of Korea and Republic of Korea. Therefore, Korean soldiers had a higher chance of contracting malaria.

Lastly, some studies reported morphology of *P. vivax* malaria and related gene sequences in the DMZ. The results support the idea that *P. vivax*-infected *Anopheles* mosquitoes came from the Democratic People’s Republic of Korea across the DMZ to the south [32, 33]. For example, two genotypes of the PvAMA-1 existed among the reemerging Korean *P. vivax* strain, which were similar to two Chinese genotypes. It could be inferred that the reemerging malaria originated from China or another Asian country, such as the Democratic People’s Republic of Korea [33].

This study had some limitations. First, the authors compared the two study areas by the number of military patients, not by the malaria prevalence rate of each area. However, the number of soldiers in the areas have been maintained at a constant rate regardless of season or weather since 2000. Therefore, the statistical differences in this study provide basic information on the changing incidence of malaria in military personnel in the two areas. Second, the AFMC counted only the number of patients who were admitted to military hospitals, and those who went to civilian hospitals were not included. Based on this limitation, the authors suggest that the exact number of malaria patients needs to be determined using malaria patients-related data of the KCDCs, the Health Insurance Review and Assessment Service, and the National Health Insurance Service, using the capture-recapture method [34–36]. Third, it was very hard to distinguish between relapse patients and reinfected patients, however, the number of such patients accounted for only 4.8% (26/531) of cases between 2006 and 2011. In addition, most of the malaria patients reported were from the army divisions located around the DMZ (19 in 26 soldiers, 73.08%). There was a considerable number of reinfected patients among the 26 cases, therefore the authors assumed that some kind of relapse or reinfection of patients would not be associated with the malaria outbreak. Lastly, the study only considered meteorological factors and areas as variables, even though there were other factors that could also have had an impact on the number of malaria patients, such as the preventive measures implemented by each military unit, the ecological relationship between vectors and hosts, regional distribution of *A. sinensis* mosquitoes, and gene typing from the Democratic People’s Republic of Korea regarding malaria [37]. Therefore, further studies should be done with these variables.

## Conclusion

Temperature, precipitation, humidity, and wind affect the incidence of malaria among military personnel stationed in certain areas. When meteorological factors and the environment are not appropriate for *A. sinensis* mosquitoes to breed and proliferate, clusters of malaria patients are found around riversides. These findings can help policymakers better understand the relationship between malaria outbreaks and meteorological factors, and establish strategies to prevent malaria infection in the military.

## Additional file

Additional file 1: Multilingual abstracts in the six official working languages of the United Nations. (PDF 295 kb)

## Abbreviations

AFMC: Armed Forces Medical Command
DMZ: Demilitarized zone
KCDC: Korea Center for Disease Control and Prevention
ROK: Republic of Korea
