# Supplementary material for: Assessing the impact of meteorological factors on malaria patients in demilitarized zones in Republic of Korea
Source: Infect Dis Poverty. 2016 Mar 8;5:20. doi: 10.1186/s40249-016-0111-3 (PMC4782315; doi:10.1186/s40249-016-0111-3)
Supplement: Additional file 1: — Multilingual abstracts in the six official working languages of the United Nations. (PDF 295 kb) [file 40249_2016_111_MOESM1_ESM.pdf]

Translation of the abstract into the six official working languages of the United Nations

تقييم تأثير العوامل الجوية على مرضى الملاريا في المناطق منزوعة السلاح في كوريا الجنوبية

سي مين هوانج، سوك چون يون، يو مي يونج، چيون يونج كوان، سو نام چو، إيون چونج چانج، ميونج-أوك كوون

ملخص

خلفية: عاد الاتجاه لإصابة المرضى العسكريين بفيروس الملاريا النشيطة إلى الظهور في جمهورية كوريا (كوريا الجنوبية) في عام 1993. وكان التفسير الشائع أن بعوضة الأنوفليس المعدية المتواجدة في كوريا الشمالية قد غزت المنطقة المنزوعة السلاح في كوريا الجنوبية. وكان الهدف من هذه الدراسة هو التحقق من العلاقة بين العوامل الجوية وعدد مرضى الملاريا في الجيش في هذه المنطقة.

الأساليب: قدر الباحثون آثار العوامل الجوية على مرضى الملاريا النشيطة في الجيش على أساس العدد الشهري لحالات الملاريا بين عامي 2006 و2011. وقد تم اختيار درجة الحرارة، والأمطار، وعمق الثلج، وسرعة الرياح، والرطوبة النسبية، ومدة تواجد أشعة الشمس، والغطاء السحابي لتكون العوامل الجوية التي يتم دراستها. تم تقييم نمط منهجي في التوزيع المكاني لحالات الملاريا باستخدام مؤشر موران. واستخدمت اختبارات جرانجر السببية ومعاملات الارتباط المقطعي لتقييم العلاقة بين العوامل الجوية ومرضى الملاريا في الجيش.

النتائج: كشف التحليل المكاني عن وجود مجموعات كبيرة من مرضى الملاريا في جيش في كوريا الجنوبية في عام 2011 (معامل موران = 0.136، القيمة الاحتمالية = 0.026). في السنوات الست للدراسة، انخفض عدد مرضى الملاريا في الجيش في باجو، ولكن زاد عدد مرضى الملاريا في الجيش في هواتشون وتشان تشون. كان المتوسط الشهري، ودرجات الحرارة القصوى والدنيا وسرعة الرياح والرطوبة النسبية عوامل للتنبؤ بمعدلات الإصابة بالملاريا لدى المرضى في الجيش في باجو. في المقابل، كانت سرعة الرياح وحدها غير قادرة على التنبؤ بإصابات الملاريا في هواتشون وتشان تشون، ومع ذلك، كانت الأمطار والغطاء السحابي قادرة على التنبؤ بإصابات الملاريا في هواتشون وتشان تشون.

الاستنتاجات: أظهرت هذه الدراسة أن عدد مرضى الملاريا في الجيش يرتبط مع العوامل الجوية. ويعزى هذا التباين في حدوث حالات الملاريا بشكل أساسي إلى الاختلافات في العوامل الجوية لأقاليم كوريا الجنوبية.

Translated from English version into Arabic by Mahmoud Sami, through

TRANSLATORS
WITHOUT BORDERS

评估气象因素对韩国非军事区域疟疾患者的影响

Se-Min Hwang, Seok-Joon Yoon, Yoo-Mi Jung, Geun-Yong Kwon, Soo-Nam Jo, Eun-Jeong Jang, Myoung-Ok Kwon

摘要

引言：1993 年在韩国军队间日疟有再现的趋势。对此通常的解释是，感染性按蚊从朝鲜进入韩国非军事区(DMZ)而引起了疟疾传播。本研究目的在于验证气象因素与该区域内军队疟疾患者数量之间的关系。

方法：基于 2006-2011 年每月疟疾病例数，估算气象因素对军队间日疟患者的影响。选取气温、降雨量、积雪深度、风速、相对湿度、日照时间和云量作为研究的气象因素，采用莫兰指数评估疟疾病例空间分布的系统模式，并用格兰杰因果检验和交叉相关系数来评价气象因素与军队疟疾病例之间的关系。

结果：空间分析显示，韩国军队疟疾病例在 2011 年有显著的集群（莫兰指数=0.136, *P* 值=0.026），六年的调查中，坡州市的军队疟疾患者数量减少，而在华川郡和春川市有所增加。以月平均值统计，风速和相对湿度可以作为坡州市的军队疟疾患者的预测因子。相比之下，单靠风速不能预测华川郡和春川市的疟疾病例数。然而，在这两个地区可以采用降雨量和云量进行预测。

结论：本研究证明军队疟疾患者数量与气象因素相关。疟疾病例出现变化主要由于韩国不同地区间气象因

素的差异所导致。

Translated from English version into Chinese by Feng Xinyu, edited by Yang Pin, through

CDC CHINESE CENTER FOR DISEASE CONTROL AND PREVENTION
NATIONAL INSTITUTE OF PARASITIC DISEASES

Évaluation de l'impact de facteurs météorologiques sur des patients atteints du paludisme dans des zones démilitarisées en Corée du Sud

Se-Min Hwang, Seok-Joon Yoon, Yoo-Mi Jung, Geun-Yong Kwon, Soo-Nam Jo, Eun-Jeong Jang, Myoung-Ok Kwon

RÉSUMÉ

Contexte : La tendance qu'ont les patients militaires à contracter le paludisme à *Plasmodium vivax* a réémergé en République de Corée (ROK) en 1993. Il a souvent été expliqué que cela était dû aux moustiques infectieux du genre *Anopheles* provenant de Corée du Nord et ayant envahi la zone démilitarisée de Corée du Sud (DMZ). L'objectif de cette étude consistait à vérifier la relation entre les facteurs météorologiques et le nombre de patients atteints du paludisme parmi les militaires dans cette région.

Méthodes : Les auteurs ont estimé les effets des facteurs météorologiques sur les patients atteints de paludisme à *Plasmodium vivax* parmi les militaires sur la base du nombre mensuel de cas de paludisme entre 2006 et 2011. La température, les précipitations, la profondeur de la neige, la vitesse du vent, l'humidité relative, la durée d'ensoleillement et la couverture nuageuse ont été sélectionnées pour servir de facteurs météorologiques à étudier. Un modèle systématique de la répartition spatiale des cas de paludisme a été évalué à l'aide d'un indice de Moran. Des tests de causalité de Granger et des coefficients de corrélation croisée ont été utilisés pour évaluer la relation entre les facteurs météorologiques et les cas de paludisme parmi les militaires.

Résultats : L'analyse spatiale a révélé l'existence d'importants groupes de patients atteints de paludisme parmi les militaires en Corée du Sud en 2011 (I de Moran = 0,136, valeur *p* = 0,026). Au cours des six ans d'analyse, le nombre de patients atteints du paludisme parmi les militaires à Paju a diminué, mais le nombre de cas de paludisme parmi les militaires de Hwacheon et de Chuncheon a quant à lui augmenté. Il a été déterminé que la moyenne mensuelle, les températures maximale et minimale, la vitesse du vent et l'humidité relative constituaient des facteurs prédictifs du paludisme chez des patients faisant partie des militaires à Paju. En revanche, la vitesse du vent à elle seule n'a pas permis de prédire le paludisme à Hwacheon et Chuncheon, mais les précipitations et la couverture nuageuse ont néanmoins permis une prédiction du paludisme à Hwacheon et Chuncheon.

Conclusions : L'étude a démontré que le nombre de patients atteints du paludisme parmi les militaires est lié aux facteurs météorologiques. La variation en termes d'occurrence de cas de paludisme était principalement attribuée à des différences en termes de facteurs météorologiques en fonction des régions de Corée du Sud.

Translated from English version into French by eric ragu, through

TRANSLATORS
WITHOUT BORDERS

Оценка воздействия метеорологических факторов на больных малярией в демилитаризованных зонах Южной Кореи

Се-Мин Хван, Сеок-Джун Юн, Ю-Ми Чон, Жеун-Юн Квон, Су-Нам Джо, Еун-Джеон Джан, Мьоунг-Ок Квон

РЕФЕРАТ

История вопроса: Тенденция заболевания военнослужащих трёхдневной малярией (*vivax* malaria) в Республике Корея (Р.К.) впервые начала проявляться в 1993 году. Обычно это объясняется вторжением в демилитаризованную зону (ДМЗ) Южной Кореи заразных малярийных комаров (*Anopheles)* из Северной Кореи. Целью данного исследования являлась проверка наличия взаимосвязи между метеорологическими факторами и количеством больных малярией военнослужащих в этой зоне.

Методы: Авторы произвели оценку воздействия метеорологических факторов на больных трёхдневной малярией (*vivax)* военнослужащих на основании помесячных данных по случаям заболевания малярией с 2006 по 2011 г. Для изучения были выбраны следующие метеорологические факторы: температура, количество атмосферных осадков, глубина снежного покрова, скорость ветра, относительная влажность, длительность солнечного сияния и облачность. Систематическая модель территориального распределения случаев заболевания малярией оценивалась с помощью индекса Морана. Для оценки взаимосвязи между метеорологическими факторами и количеством заболевших малярией военнослужащих использовались тесты Грейнджера и коэффициенты взаимной корреляции.

Результаты: Пространственный анализ выявил значительные группы больных малярией военнослужащих в Южной Корее в 2011 (индекс Морана = 0.136, Значение *p*- = 0.026). За шесть изученных лет количество больных малярией военнослужащих в Пхаджу уменьшилось, но количество больных малярией военнослужащих в городах Хвачеон и Чунчеон увеличилось. Было установлено, что предсказывающими факторами заболевания военнослужащих малярией в Пхаджу являются среднемесячная, максимальная и минимальная температура, скорость ветра и относительная влажность. В тоже время оказалось невозможным предсказать заболеваемость малярией только на основании скорости ветра в городах Хвачеон и Чунчеон, однако, количество осадков и облачность в этой зоне являлись предсказывающими факторами.

Выводы: Данное исследование доказало наличие соотношения между количеством заболевающих малярией военнослужащих и метеорологическими факторами. Различия в частоте заболеваемости малярией в основном приписывается различиям в метеорологических факторах в разных регионах Южной Кореи.

Translated from English version into Russian by Alena Hrybouskaya, through

Evaluación del impacto de factores meteorológicos sobre pacientes con malaria en zonas demilitarizadas de Corea del Sur

Se-Min Hwang, Seok-Joon Yoon, Yoo-Mi Jung, Geun-Yong Kwon, Soo-Nam Jo, Eun-Jeong Jang, Myoung-Ok Kwon

RESUMEN

Antecedentes: En el año 1993 se produjo en la República de Corea un resurgimiento de la tendencia de pacientes militares a sufrir infección por el virus *vivax* de la malaria. La explicación normal ha sido que mosquitos *Anopheles* de Corea del Norte portadores de la infección invadieron la zona demilitarizada de Corea del Sur (ZDC). El propósito de este estudio fue verificar la relación entre los factores meteorológicos y la cantidad de pacientes en el ejército con malaria en dicha región.

Métodos: Los autores estimaron los efectos de los factores meteorológicos en pacientes del ejército con malaria *vivax* en base a la cantidad mensual de casos de malaria entre los años 2006 y 2011. Se seleccionaron temperatura, precipitación, profundidad de la nieve, velocidad del viento, humedad relativa, duración de luz solar y nubosidad como los factores meteorológicos a estudiarse. Se utilizó el índice de Moran para evaluar un patrón sistemático en la distribución espacial de casos de malaria. Se utilizaron pruebas de causalidad de Granger y coeficientes de correlación cruzada para evaluar la relación entre los factores meteorológicos y los pacientes en el ejército con malaria.

Resultados: El análisis espacial reveló grupos significativos de pacientes con malaria en el ejército en Corea del Sur en el año 2011 (I. de Moran=0,136, valor p=0,026). En los seis años investigados, la cantidad de pacientes con malaria en el ejército en Paju disminuyó, pero la cantidad de pacientes con malaria en el ejército en Hwacheon y Chuncheon aumentó. Se encontró que la temperatura promedio mensual, las temperaturas máximas y mínimas; la velocidad del viento y la humedad relativa fueron factores predictores de malaria en los pacientes en el ejército en Paju. Por el contrario, la velocidad del viento por sí sola, no podía predecir malaria en Hwacheon y Chuncheon. Sin embargo, la precipitación y la nubosidad pudieron predecir malaria en Hwacheon y Chuncheon.

Conclusiones: Este estudio demostró que la cantidad de pacientes con malaria en el ejército está asociada a factores meteorológicos. La variación en ocurrencia de casos de malaria se atribuyó principalmente a diferencias en los factores meteorológicos en las distintas regiones de Corea del Sur.

Translated from English version into Spanish by Maria Alejandra Aguada, through
